# Parental and peer social support is associated with healthier physical activity behaviors in adolescents: a cross-sectional analysis of Texas School Physical Activity and Nutrition (TX SPAN) data

**DOI:** 10.1186/s12889-019-7001-0

**Published:** 2019-05-27

**Authors:** Amier Haidar, Nalini Ranjit, Natalie Archer, Deanna M. Hoelscher

**Affiliations:** 1 Michael & Susan Dell Center for Healthy Living, UTHealth School of Public Health, 1616 Guadalupe St, Suite 6.300, Austin, TX 78701 USA; 2 Texas Department of State Health Services, 1100 W 49th Street, Austin, TX 78756 USA

**Keywords:** Parental and peer support, Adolescents, Physical activity, Screen-time, Survey research

## Abstract

**Background:**

Parental and peer support can influence children’s physical activity; however, these associations have not been fully examined in a multi-ethnic population across early and late adolescence. The objective of this study was to examine associations between perceived parental/peer social support, perceived parental disapproval for not exercising, and physical activity/screen time behaviors among a multi-ethnic sample of adolescents.

**Methods:**

The Texas School Physical Activity and Nutrition (TX SPAN) survey is a cross-sectional statewide probability-based survey, used to assess obesity-related behaviors such as diet and physical activity. The SPAN 2009–2011 study measured 8th and 11th grade students using a self-report questionnaire with established psychometric properties, along with objectively measured height and weight. Associations were examined using multiple logistic and linear regression.

**Results:**

For every 1-point increase in parental physical activity support, adolescents had 1.14 higher odds of engaging in five or more days of moderate physical activity per week (*p* < 0.001), and 1.12 higher odds of engaging in three or more days of vigorous physical activity per week (*p* < 0.001). For every 1-point increase in peer physical activity support, adolescents had 1.17 higher odds of engaging in five or more days of moderate physical activity per week (*p* < 0.001), and 1.15 higher odds of engaging in three or more days of vigorous physical activity per week (*p* < 0.001).

**Conclusions:**

Parental and peer social support is associated with positive physical activity behaviors in adolescents. Strategies to focus on parent and peer support should be integral to intervention programs designed to increase physical activity in adolescents in middle and high schools.

## Background

A multitude of factors have been shown to influence childhood health, including physical activity. The USDHHS Physical Activity Guidelines recommend that children engage in moderate to vigorous intensity activity for at least 60 min a day [1]. This requirement is largely unmet by adolescents in Texas, with only 25% of adolescents engaging in moderate to vigorous physical activity for at least 60 min every day [2]. This is important to note because obesity has been associated with decreased physical activity and increases in sedentary activities, such as screen time; increased physical activity has also been associated with numerous health benefits in regards to depressive symptoms, metabolic syndrome, high blood pressure, and cholesterol [3–5].

Parents have been shown to have a strong influence on adolescent physical activity. A strong association between parental support and the level of child physical activity was reported, which further showed that effective support can be operationalized as encouragement, involvement, or facilitation [6]. A small association was found between parent physical activity and child physical activity, with greater physical activity in parents being associated with greater physical activity in children [7]. A noteworthy finding was that overall parental support had a moderate effect size on child physical activity, and of the individual behaviors consisting of parental support, parental encouragement had the greatest effect [7]. Parental support significantly predicted adolescent physical activity, family cohesion, and parent-child communication [8]. Overall parent support determined whether adolescents met the recommended physical activity guidelines 1 year later; in particular, parental engagement, a component of parent support, was associated with meeting the guidelines as well [8]. As with physical activity, sedentary time of parents is associated with the sedentary time of daughters: that high parental TV viewing increased the risk that children spent more than 4 h per day watching TV [9]. Taken together, these studies demonstrate the effects parents can have on either the physical activity levels or screen time of their children.

Peers also can have a strong influence on adolescent decision-making. Many studies have examined the influence of peers on their friends’ physical activity behaviors [10]. One study found that peers had a significant effect on each other’s levels of physical activity [11]. Another study conducted among African American adolescents found that boys received more support from their peers to engage in physical activity than did girls, and that girls reported receiving no peer support for physical activity [12]. Among 5th–7th grade girls, parents (especially mothers), rather than peers, were found to be the primary means of social support for physical activity [13]. Peer support during physical activity was associated with increased physical activity, and this association may be greater for overweight adolescents [14].

Despite the number of studies that have elucidated the effects of parental and peer support on physical activity, few studies have included diverse populations and no studies have included both males and females at two distinct developmental levels – middle and high school adolescents [15–17]. Examining two distinct age groups in an ethnically diverse population will allow for further understanding of the associations between parental/peer support for physical activity, as well as parental disapproval for not exercising and physical activity behaviors in adolescents. This will allow for the development of strategies and programs that can effectively target parents and peers, to increase physical activity and decrease screen-time in adolescents. The objective of this study was to investigate associations between perceived parental/peer social support, perceived parental disapproval for not exercising, and adolescents’ physical activity and screen time behaviors, utilizing Texas School Physical Activity and Nutrition (SPAN) data collected from 8th and 11th grade students in 2009–2011.

## Methods

### Study design

The study design was a cross-sectional survey, which assessed obesity and health behaviors such as diet and physical activity [18]. Texas SPAN used a stratified, multistage sampling plan that provides state-representative data for elementary (4th grade), and high school (8th and 11th grade) students; this study only uses data from high school students. Study methods are briefly described below, but further descriptions of the SPAN study and detailed methodology are presented elsewhere [18, 19]. The Committee for the Protection of Human Subjects at the University of Texas Health Science Center at Houston (UTHealth) (HSC-SPH-18-0432), the Texas Department of State Health Services Institutional Review Board, and local school district review committees have reviewed and approved this study.

### Participants

The Texas SPAN 2009–2011 survey included 6716 8th and 11th grade students (*n* = 3931 8th grade and *n* = 2.785 11th grade), to capture distinct developmental stages of early and late adolescence. The data for SPAN 2009–2011 were collected over the 2009–2010 and 2010–2011 school years. The Texas Education Agency data from 2009 to 2010 were used to provide the sampling frame for the study, e.g., the weighting of the data was indexed for the Texas school-aged population for those years.

### Data collection

The SPAN survey is a self-administered survey questionnaire. SPAN items, which include demographic questions as well as questions about physical activity, have been previously tested and assessed for reproducibility [20].

### Demographics

Race/ethnicity consisted of African American, Hispanic/Latino, or White/Other. Other accounted for 3% of the Texas population and included non-Hispanic white, Native American, Asian, Pacific Islander, or “other” [18]. The percentage of students who received free and reduced-price lunches was used to determine socioeconomic status. To assess parent education level, adolescents were asked two survey questions about the highest level of education their parents had completed. Height and weight were measured by study staff that were trained in proper measurement and certified before field data collection, using a stadiometer (PE-AIM-101) and scale (Tanita BWB-800S). Scales were calibrated before every measurement day, and quality control was also conducted by re-weighing a random selection of 5.6% of measurements. BMI was calculated and used to determine weight status, as categorized by the CDC BMI growth charts.

### Measures

Measurements analyzed in this study included parental and peer physical activity support, perceived parental disapproval for not exercising, and adolescent physical activity measures, including: amount of moderate physical activity, amount of vigorous physical activity, amount of screen-time, and an overall physical activity score.

In the SPAN survey, two types of physical activity social support were examined: parental support and peer support. Measures for parental and peer support were developed from previously validated scales [15, 16, 21, 22]. Three questions were used to measure parental support: I have parents or guardians who … (a) “want me to exercise or be physically active”, (b) “exercise with me”, and (c) “spend time teaching me to play a sport or do a physical activity”. Similar questions were used to measure peer support with the stem of the above questions edited to “I have friends who …” . The responses to each of the three questions were never, almost never, sometimes, almost always, and always. Each question had responses ranging from 0 to 4, which were then summed to create a total overall range from 0 (least support) to 12 (most support).

Perceived parental disapproval for not exercising was assessed with the question, “How upset would your parents feel if they found out you were not exercising?”, which was adapted from a question used in a previous study [23] using similar questions that assessed adolescent perceptions of parent anger in response to drug use [24]. The responses included 1) not at all upset, 2) a little upset, 3) pretty upset, and 4) very upset. These responses were then dichotomized into upset (responses to answer choices 2, 3, 4) and not upset (responses to answer choice 1).

Moderate and vigorous physical activity were measured by two questions similar to those used in the Youth Risk Behavior Survey [25]: “During the past 7 days, on how many days were you physically active for a total of at least 60 minutes per day? (Add up all the time you spent in any kind of physical activity that increased your heart rate and made you breathe hard some of the time.)” and “On how many of the past 7 days did you exercise or take part in physical activity that made your heart beat fast and made you breathe hard for at least 20 minutes? (For example: basketball, soccer, running or jogging, fast dancing, swimming laps, tennis, fast bicycling, or similar aerobic activities)”. The responses to each question were collapsed based on physical activity recommendations by the USDHHS Physical Activity Guidelines [1]. Moderate physical activity was collapsed into five or more days and less than five. Vigorous physical activity was collapsed into three or more days and less than three.

Screen-time was measured by three questions: “How many hours per day do you usually watch TV, DVDs, or movies away from school?”, “How many hours per day do you usually spend on a computer away from school?”, and “How many hours per day do you usually spend playing video games like Nintendo® Wii or DS, Sega®, PlayStation®, Xbox®, GameBoy®, or arcade games away from school?”. The responses to these questions were combined to create a scale and collapsed into two groups, based on screen-time recommendations from the American Academy of Pediatrics: 1) those that spent less than 2 h per day, and 2) those that spent 2 or more hours per day engaging in screen-time [26].

A SPAN Physical Activity Index (SPAI) was created based on methodology for the SPAN Healthy Eating Index [27], which earlier studies have determined has adequate predictive validity, and varies reliably with other measures [27]. Physical activity behaviors for the SPAI consisted of responses to questions asking about moderate physical activity for at least 60 min per day, vigorous physical activity for at least 20 min per day, and strength training. Screen-time behaviors included responses to survey items assessing hours watching TV, spent on the computer, and playing video games. To calculate this score, the responses to each question were dichotomized into “0 = No” or “1 = Yes”, with “no” indicating that the adolescent did not engage in these physical/screen-time -related activities, while ‘yes’ indicated that they did. Summary scores for screen-time and physical activity behaviors were created separately and each score was standardized from 0 to 100 points. The screen-time index was then subtracted from the physical activity index to create an overall physical activity score, with a range from − 100 to 100, where higher positive scores indicated more physical activity and less screen-time, and negative scores indicated more screen-time than physical activity.

### Statistical analysis

Statistical analyses were performed with STATA 13.0 (College Station, TX), using appropriate sampling weights for the state. Weighted descriptive statistics were calculated, and Pearson chi-square tests and t-tests were used to determine differences in the frequency or mean of parental/peer support, and physical activity variables. Relations between parental/peer support and physical activity variables were examined using a multiple logistic regression model. The relations between parental/peer support and overall physical activity score was examined using a linear regression model. Covariates included grade, gender, ethnicity, weight status, socioeconomic status, and parents’ education level.

## Results

The demographics of the study population are presented in Table 1. The sample was split fairly evenly by grade, and consisted of 40% White/Other, 15% African American, and 45% Hispanic students. Most of the students in the sample were at a healthy weight (62%), while 16% were classified as having overweight and 22% were classified as having obesity. The majority of parents had a high school education or less (60%). The majority of students went to schools with a socioeconomic status in either the lowest (37%) or middle tertile (38%). Data were also stratified by gender; demographic findings were similar between boys and girls.

**Table 1:** Demographic characteristics of the school physical activity and nutrition (SPAN) 2009–2011 8th and 11th grade population

	Total (*n* = 6716)^1^	Boy (*n* = 3251)	Girl (*n* = 3465)
Age, Years, Mean	14.88	14.9	14.8
Grade, %			
8th	53.3	53.6	53.0
11th	46.7	46.4	47.0
Ethnicity, %			
White/Other	39.8	40.1	39.6
African-American	14.6	14.5	14.7
Hispanic	45.6	45.5	45.7
BMI Class^1^, %			
Healthy Weight	62.0	57.6	66.4
Overweight	15.7	16.1	15.3
Obese	22.3	26.3	18.3
Parent Education, %			
Some college or more	40.3	40.7	39.9
High school or less	59.7	59.3	60.1
Socioeconomic Status, %			
Highest Tertile	25.0	24.2	25.9
Middle Tertile	38.2	38.2	38.1
Lowest Tertile	36.8	37.7	35.9

Note: 1. Overweight is defined as >85th and < 95th percentile, while Obese is defined as ≥95th percentile

^1^This number represents 3931 8th grade students (representing 310,045 8th grade students in Texas) and 2.785 11th grade students (representing 272,122 11th grade students in Texas)

Frequencies of parent and peer physical activity support are shown in Table 2. Overall, 47% of adolescents reported having parents that disapproved of them not exercising. Only 38% of eleventh graders reported having parents that disapproved of them not exercising, compared with 54% of 8th graders (*p* < 0.001). A lower proportion of adolescents classified as healthy weight indicated having parents that disapproved of them not exercising (41%), compared with both overweight and obese adolescents (56% for both, *p* < 0.001), with no differences by gender.

**Table 2:** Frequencies of parent and peer physical activity support, by demographic characteristics, TX SPAN 8th and 11th grade students, 2009–2011

	Perceived Parental Disapproval for not exercising		Parental Physical Activity Support	Peer Physical Activity Support	Screen-time		Moderate Physical Activity		Vigorous PA		Physical Activity Score
	Not Upset	Upset	Mean	Mean	< 2 h/day	≥ 2 h/day	Less than 5 days/wk	5 or more days/wk	Less than 3 days/wk	3 or more days/wk	Mean
Gender, %											
Male	50.4	49.6	5.6	6.4	14.8***	85.2	37.8**	62.2	21.5***	78.5	−13.23
Female	56.0	44,0	5.44	5.6	24.4	75.6	51.4	48.6	32.7	67.3	−12.36
Grade, %											
8th	45.9***	54.1	5.9	5.9	14.7	85.3	43.8	56.2	26.9	73.1	−12.97
11th	62.1	37.9	5.0	6.1	25.1	74.9	45.3	54.7	27.1	72.9	−12.62
Ethnicity, %											
White/Other	55.8	44.2	5.3	5.8	23.2	76.8	41.4*	58.6	23.1	76.9	−10.69
African-American	61.0	39.0	6.2	6.8	13.6	86.4	40.3	59.7	26.6	73.4	−11.56
Hispanic	48.3	51.7	5.5	5.8	18.2	81.8	48.5	51.5	30.5	69.5	−15.08
BMI Classification, %											
Healthy Weight	58.8***	41.2	5.2	5.9	20	80	40.4**	59.6	25.5	74.5	−10.63
Overweight	43.7	56.3	5.9	6.1	19.4	80.6	48.0	52.0	27.2	72.8	−13.48
Obese	44.2	55.8	5.9	6.1	18.3	81.7	53.4	46.6	30.9	69.1	−18.21
Parent Education, %											
Some College or more	52.5	47.5	6.1	6.3	15.9	84.1	39.9*	60.1	25.4	74.6	−10.35
High School or less	52.1	47.9	5.2	5.8	14.5	85.5	50.7	49.3	29.7	70.3	−14.31
Socioeconomic Status, %											
Highest Tertile	48.5	51.5	5.4	5.8	17.9	82.1	54.7*	45.3	33.5*	66.5	−16.67
Middle Tertile	56.5	43.5	5.3	6.1	23.8	76.2	39.1	60.9	23.5	76.5	−9.05
Lowest Tertile	53.2	46.8	5.7	6.0	15.5	84.5	43.1	56.9	26.1	73.9	−13.75
TOTAL	53.2	46.8	5.7	6.4	19.5	80.5	44.5	55.5	27.0	73.0	−12.81

Note: Pearson chi-square tests and t-tests were calculated to determine differences by categorical and continuous demographic variables, respectively. *P < 0·05, **P < 0·01, ***P < 0·001

Significant differences in levels of screen-time and physical activity were evident by gender. Over two-thirds of males (85%) reported engaging in two or more hours of screen-time per day, compared with 75.5% of females (*p* < 0.001). A greater proportion of males than females also reported engaging in five or more days of 60 min of moderate physical activity (62% vs 49%, *p* < 0.01) and three or more days of 20 min of vigorous physical activity per week (79% vs. 67%, *p* < 0.001). Three-quarters of all White/Other adolescents (76.8%) engaged in two or more hours of screen-time per day, compared with 86.4 and 81.8% of African-American and Hispanic adolescents, respectively (approaching significance *p* = 0.06).

Parental disapproval of not exercising was a strong predictor of parental support for physical activity. Adolescents who reported having parents that disapproved of them not exercising had a higher parental support for physical activity score (*p* < 0.001).

Table 3 presents logistic regression odds ratios and linear regression beta coefficients comparing associations between demographics of the study population and perceived parental/peer support for physical activity, as well as perceived parental disapproval for not exercising. Eleventh grade students had 1.9 times lower odds of having parents that disapproved of them not, compared to eighth grade students (*p* < 0.001). In addition, eleventh graders had a lower mean parental physical activity support score than eighth graders (0.84 points lower, *p* < 0.01). Adolescents who had overweight or obesity had 1.8 times higher odds of having parents that disapproved of them not exercising, compared with adolescents classified as healthy (*p* = 0.004, *p* < 0.001). In addition, adolescents who had overweight or obesity also had parental physical activity support scores that were 0.67 and 0.71 points higher than adolescents classified as healthy, respectively (*p* < 0.05). Parental and peer physical activity support scores were 0.84 and 0.85 points higher among African-American adolescents than among White/Other adolescents, respectively (*p* < 0.01). Adolescents whose parents had some college education or more had a mean parental physical activity support score that was 0.89 points higher than among adolescents whose parents had a high school education or less (*p* = 0.001). Lastly, the mean peer physical activity support score among girls was − 0.74 points lower than among boys (*p* < 0.05).

**Table 3:** Linear and Logistic Regressions between demographic variables and independent physical activity variables, TX SPAN 8th and 11th grade students, 2009–2011

	Perceived Parental Disapproval for not exercising		Parental Physical Activity Support		Peer Physical Activity Support	
	Odds Ratio	*P*-value	Beta Coeff	*P*-value	Beta Coeff	*P*-value
Gender^a^						
Girl	0.83 (0.67, 1.03)	0.082	−0.10 (−0.50, 0.30)	0.615	−0.74 (−1.36, − 0.12)	0.021
Grade^b^						
11th	0.53 (0.39, 0.70)	*P* < 0.001	−0.84 (−1.35, − 0.32)	0.002	0.22 (−0.38, 0.82)	0.465
Ethnicity^c^						
African-American	0.80 (0.54, 1.18)	0.249	0.84 (0.31, 1.37)	0.002	0.85 (0.22, 1.48)	0.008
Hispanic	1.26 (0.86, 1.82)	0.231	0.32 (−0.26, 0.90)	0.284	0.07 (−0.49, 0.64)	0.802
BMI Class^d^						
Overweight	1.78 (1.21, 2.61)	0.004	0.67 (0.12, 1.21)	0.017	0.16 (−0.42, 0.73)	0.591
Obese	1.81 (1.29, 2.54)	0.001	0.71 (0.08, 1.33)	0.027	0.09 (−0.61, 0.79)	0.802
Parent Education^e^						
Some College or more	1.03 (0.78, 1.36)	0.846	0.89 (0.39, 1.39)	0.001	0.47 (−0.12, 1.05)	0.117
Socioeconomic Status^f^						
Middle Tertile	0.87 (0.62, 1.21)	0.389	−0.30 (− 0.86, 0.267)	0.298	0.15 (− 0.67, 0.97)	0.721
Highest Tertile	0.94 (0.64, 1.40)	0.775	−0.33 (− 0.91, 0.26)	0.267	0.26 (− 0.78, 0.84)	0.949

Note: Confidence intervals for odds ratios are in parenthesis. Referents were ^a^Boy, ^b^8th, ^c^White/Other, ^d^Healthy Weight, ^e^High school or less, ^f^Lowest Tertile

Table 4 presents logistic regression odds ratios and linear regression beta coefficients showing levels of association between parental/peer physical activity support, parental disapproval for not exercising and the physical activity response variables. For every 1-point increase in parental support for physical activity, adolescents had 1.1 lower odds of engaging in two or more hours of screen-time per day (*p* < 0.05), 1.14 higher odds of engaging in five or more days of moderate physical activity per week (*p* < 0.001), 1.12 higher odds of engaging in three or more days of vigorous physical activity per week (*p* < 0.001), and had a physical activity score that was 2.4 points higher (*p* < 0.001). For every 1-point increase in peer support for physical activity, adolescents had 1.1 lower odds of engaging in two or more hours of screen-time per day (*p* < 0.001), 1.17 higher odds of engaging in five or more days of moderate physical activity per week (*p* < 0.001), 1.15 higher odds of engaging in three or more days of vigorous physical activity per week (*p* < 0.001), and had a physical activity score that was 2.91 points higher (*p* < 0.001).

**Table 4:** Linear and Logistic Regression for associations between covariates and physical activity behaviors, TX SPAN 8th and 11th grade students, 2009–2011

	Screen-time		Moderate Physical Activity		Vigorous Physical Activity		Physical Activity Score	
	Odds Ratio	*P*-value	Odds Ratio	*P*-value	Odds Ratio	*P*-value	Beta Coefficient	*P*-value
Perceived Parental Disapproval for not exercising^a^								
Upset	0.72 (0.39, 1.33)	0.296	1.12 (0.81, 1.55)	0.497	1.25 (0.90, 1.74)	0.186	3.43 (−1.91, 8.77)	0.206
Parental Physical Activity Support								
	0.92 (0.86, 0.98)	0.014	1.14 (1.07, 1.21)	*P* < 0.001	1.12 (1.10, 1.17)	*P* < 0.001	2.40 (1.27, 3.54)	*P* < 0.001
Peer Physical Activity Support								
	0.93 (0.87, 0.98)	0.019	1.17 (1.13, 1.22)	*P* < 0.001	1.15 (1.10, 1.20)	*P* < 0.001	2.91 (2.01, 3.81)	*P* < 0.001
Gender^b^								
Girl	0.44 (0.33, 0.62)	*P* < 0.001	0.53 (0.37, 0.74)	*P* < 0.001	0.53 (0.42, 0.67)	*P* < 0.001	0.14 (−5.86, 6.14)	0.963
Grade^c^								
11th	0.68 (0.44, 1.05)	0.082	0.77 (0.57, 1.04)	0.087	0.84 (0.60, 1.18)	0.32	−0.24 (,-8.66, 8.18)	0.956
Ethnicity^d^								
African-American	1.84 (0.96, 3.55)	0.068	1.07 (0.77, 1.50)	0.669	0.83 (0.54, 1.27)	0.391	−2.04 (−12.40, 8.32)	0.698
Hispanic	1.74 (0.99, 3.08)	0.054	0.87 (0.69, 1.10)	0.239	0.75 (0.49, 1.13)	0.165	−2.91 (−10.62, 4.80)	0.457
BMI Class^e^								
Overweight	0.94 (0.64, 1.39)	0.762	0.69 (0.53, 0.92)	0.011	0.90 (0.60, 1.33)	0.58	−2.69 (−7.80, 2.62)	0.319
Obese	0.81 (0.55, 1.21)	0.304	0.57 (0.40, 0.83)	0.004	0.75 (0.52, 1.10)	0.132	−7.23 (−15.28, 0.83)	0.078
Parent Education^f^								
Some College or more	1.14 (0.79, 1.64)	0.479	1.37 (0.96, 1.96)	0.083	1.10 (0.71, 1.67)	0.698	3.57 (−5.31, 12.44)	0.428
Socioeconomic Status^g^								
Middle Tertile	1.04 (0.53, 2.09)	0.892	1.214 (0.85, 1.71)	0.288	1.05 (0.69, 1.59)	0.819	6.18 (−4.90, 17.27)	0.272
Highest Tertile	0.89 (0.49, 1.57)	0.676	0.75 (0.53, 1.06)	0.106	0.77 (0.50, 1.20)	0.25	−0.50 (−9.27, 8.28)	0.911

Note: Confidence intervals for odds ratios are in parenthesis. Referents were ^a^Not upset, ^b^Boy, ^c^8th, ^d^White/Other, ^e^Healthy Weight, ^f^High school or less, ^g^Lowest Tertile

## Discussion

This study adds to the literature by determining physical activity patterns associated with parental and peer support, using a large and diverse sample of 8th and 11th grade adolescents. Study results indicate that parents and peers have a significant effect on the physical activity of adolescents in Texas. Adolescents who received more parental and peer support for physical activity were more physically active than adolescents who received less support.

It is known that physical activity is associated with a lower risk of obesity, while increased screen-time is associated with a greater risk [3, 4]. Our study found that adolescents in Texas who had parents that disapproved of them not exercising and who received parental support for physical activity had lower odds of engaging in two or more hours of screen-time per day and higher odds of engaging in both five or more days of moderate physical activity per week and three or more days of vigorous physical activity per week. These results are consistent with previous studies that showed that parental support influenced child and adolescent physical activity and sedentary time [6–9, 28, 29].

These findings also demonstrated that peers strongly influenced adolescent physical activity and screen-time behaviors. Adolescents who received peer support showed lower odds of engaging in two or more hours of screen-time per day and higher odds of engaging in five or more days of moderate physical activity, as well as three or more days of vigorous physical activity per week. These results are supported by other literature that showed similar correlations between peer support and physical activity levels [10, 11, 14]. Our study also found that boys had higher odds of receiving physical activity support from their peers than did girls. These findings are similar to another study that found that boys received more support from their peers to engage in physical activity [12].

This research expands on previous studies by examining the effects of peers on screen-time related behavior, since peer support was associated with lower odds of screen-time per day. It is also noteworthy that, in our study, adolescents reported receiving higher levels of physical activity support from their peers than from their parents; this is in contradiction to a study conducted by Ling and colleagues which observed that parents were the primary means of social support for physical activity [13]. These differing results might be explained by differences in study populations. The sample used by Ling and co-researchers consisted of only girls in the 5th–7th grade, while our sample consisted of boys and girls in the 8th and 11th grade.

Results comparing gender and physical activity showed girls had lower odds of engaging in screen-time and physical activity. This finding is consistent with results from several other studies [4, 30–35].

Study findings showed that adolescents with overweight or obesity had lower odds of engaging in five or more days of moderate physical activity per week than adolescents with healthy weights. This is consistent with multiple studies which have found that physical activity is associated with lower weight status [4, 31, 32]. Our results also revealed that adolescents with overweight and obesity more frequently had parents that disapproved of them not exercising and had higher odds of perceiving parental disapproval for not exercising. This could indicate that perhaps parents disapproved of physical inactivity in an effort to curb overweight or obesity by the parents.

Limitations of this study included the study design, self-reported measures, and the time period of the measures. Causal inferences cannot be made due the cross-sectional study design of the study; however, this design did give us a large and diverse statewide sample with which to examine these associations and to help develop intervention strategies for a broad range of diverse populations. Since data were self-reported, measures could be subject to biases, such as underreporting, social biases, or missing data; however, the reliability and validity of SPAN questionnaire items have been evaluated in similar diverse adolescent populations [20]. The parental and peer support scales to our knowledge have not been specifically validated in adolescent males. The parental support measure also did not distinguish between mothers and fathers, so we were unable to draw conclusions about whether one parent’s support had a greater impact compared to the other. The age of the dataset is another limitation, as the data are from 2009 to 2011, and secular trends might influence the associations found in this study.

## Conclusion

Based on results from our study, programs should be created and focused on targeting parents and peers to increase their physical activity support for adolescents. The adolescent age group is crucial for the development of targeted intervention and educational programs because they are developing habits that can have a sustained impact on their future [36, 37]. Therefore, it is necessary to ensure that adolescents develop healthy lifestyle habits, such as regularly engaging in physical activity that can carry over into adulthood.

## Data Availability

The datasets used and analyzed during the current study are available from the corresponding author on reasonable request.

## Abbreviations

SPAN: School Physical Activity and Nutrition

## References

[1] Physical Activity Guidelines Advisory Committee: Physical activity guidelines advisory committee report, 2008. *Washington,* DC: US Department of Health and Human Services 2008, 2008:A1-H14.

[2] Kann L, McManus T, Harris WA, Shanklin SL, Flint KH, Queen B, Lowry R, Chyen D, Whittle L, Thornton J (2018). Youth risk behavior surveillance—United States, 2017. MMWR Surveill Summ.

[3] Zhang G, Wu L, Zhou L, Lu W, Mao C (2016). Television watching and risk of childhood obesity: a meta-analysis. Eur J Pub Health.

[4] Nemet D (2016). Childhood obesity, physical activity, and exercise. Pediatr Exerc Sci.

[5] Janssen I, LeBlanc AG (2010). Systematic review of the health benefits of physical activity and fitness in school-aged children and youth. Int J Behav Nutr Phys Act.

[6] Gustafson SL, Rhodes RE (2006). Parental correlates of physical activity in children and early adolescents. Sports Med.

[7] Yao CA, Rhodes RE (2015). Parental correlates in child and adolescent physical activity: a meta-analysis. Int J Behav Nutr Phys Act.

[8] Ornelas IJ, Perreira KM, Ayala GX (2007). Parental influences on adolescent physical activity: a longitudinal study. Int J Behav Nutr Phys Act.

[9] Jago R, Fox KR, Page AS, Brockman R, Thompson JL (2010). Parent and child physical activity and sedentary time: do active parents foster active children?. BMC Public Health.

[10] Chung SJ, Ersig AL, McCarthy AM (2017). The influence of peers on diet and exercise among adolescents: a systematic review. J Pediatr Nurs.

[11] Finnerty T, Reeves S, Dabinett J, Jeanes YM, Vögele C (2010). Effects of peer influence on dietary intake and physical activity in schoolchildren. Public Health Nutr.

[12] St. George SM, Wilson DK (2012). A qualitative study for understanding family and peer influences on obesity-related health behaviors in low-income African-American adolescents. Child Obes.

[13] Ling J, Robbins LB, Resnicow K, Bakhoya M (2014). Social support and peer norms scales for physical activity in adolescents. Am J Health Behav.

[14] Fitzgerald A, Fitzgerald N, Aherne C (2012). Do peers matter? A review of peer and/or friends’ influence on physical activity among American adolescents. J Adolesc.

[15] Dishman RK, Hales DP, Sallis JF, Saunders R, Dunn AL, Bedimo-Rung AL, Ring KB (2009). Validity of social-cognitive measures for physical activity in middle-school girls. J Pediatr Psychol.

[16] Donnelly R, Springer A (2015). Parental social support, ethnicity, and energy balance-related behaviors in ethnically diverse, low-income, urban elementary schoolchildren. J Nutr Educ Behav.

[17] Springer AE, Kelder SH, Byrd-Williams CE, Pasch KE, Ranjit N, Delk JE, Hoelscher DM (2013). Promoting energy-balance behaviors among ethnically diverse adolescents: overview and baseline findings of the Central Texas CATCH middle school project. Health Educ Behav.

[18] Hoelscher DM, Day RS, Lee ES, Frankowski RF, Kelder SH, Ward JL, Scheurer ME (2004). Measuring the prevalence of overweight in Texas schoolchildren. Am J Public Health.

[19] Hoelscher DM, Kelder SH, Pérez A, Day RS, Benoit JS, Frankowski RF, Walker JL, Lee ES (2010). Changes in the regional prevalence of child obesity in 4th, 8th, and 11th grade students in Texas from 2000–2002 to 2004–2005. Obesity.

[20] Hoelscher DM, Day RS, Kelder SH, Ward JL (2003). Reproducibility and validity of the secondary level school-based nutrition monitoring student questionnaire. Journal of American Dietic Association.

[21] Prochaska JJ, Rodgers MW, Sallis JF (2002). Association of parent and peer support with adolescent physical activity. Research Quarterly of Exercise and Sport.

[22] Sallis JF, Taylor WC, Dowda M, Freedson PS, Pate RR (2002). Correlates of vigorous physical activity for children in grades 1 through 12: comparing parent-reported and objectively measured physical activity. Pediatr Exerc Sci.

[23] Windle M, Brener N, Cuccaro P, Dittus P, Kanouse DE, Murray N, Wallander J, Schuster MA (2010). Parenting predictors of early-adolescents’ health behaviors: simultaneous group comparisons across sex and ethnic groups. Journal of Youth and Adolescence.

[24] Parsai M, Voisine S, Marsiglia FF, Kulis S, Nieri T (2009). The protective and risk effects of parents and peers on substance use, attitudes, and behaviors of Mexican and Mexican American female and male adolescents. Youth Soc.

[25] Eaton DK, Kann L, Kinchen S, Shanklin S, Ross J, Hawkins J, Harris WA, Lowry R, McManus T, Chyen D, Lim C, Brener ND, Wechsler H (2008). Centers for Disease Control and Prevention (CDC): youth risk behavior surveillance--United States, 2007. MMWR Surveillance Summaries.

[26] Strasburger VC, Council on Communications and Media (2011). Children, adolescents, obesity, and the media. Pediatrics.

[27] Ranjit N, Wilkinson AV, Lytle LM, Evans AE, Saxton D, Hoelscher DM (2015). Socioeconomic inequalities in children’s diet: the role of the home food environment. Int J Behav Nutr Phys Act.

[28] Eisenberg ME, Larson NI, Berge JM, Thul CM, Neumark-Sztainer D (2014). The home physical activity environment and adolescent BMI, physical activity, and TV viewing: disparities across a diverse sample. J Racial Ethn Health Disparities.

[29] Trost SG, Loprinzi PD (2011). Parental influences on physical activity behavior in children and adolescents: a brief review. Am J Lifestyle Med.

[30] Groth SW, Rhee H, Kitzman H (2016). Relationships among obesity, physical activity and sedentary behavior in young adolescents with and without lifetime asthma. J Asthma.

[31] Butte NF, Puyau MR, Adolph AL, Vohra FA, Zakeri I (2007). Physical activity in nonoverweight and overweight Hispanic children and adolescents. Med Sci Sports Exerc.

[32] D'Addesa D, D'Addezio L, Martone D, Censi L, Scanu A, Cairella G, Spagnolo A, Menghetti E (2010). Dietary intake and physical activity of normal weight and overweight/obese adolescents. International Journal of Pediatrics.

[33] Belcher BR, Berrigan D, Dodd KW, Emken BA, Chou CP, Spruijt-Metz D (2010). Physical activity in US youth: effect of race/ethnicity, age, gender, and weight status. Med Sci Sports Exerc.

[34] Butt J, Weinberg RS, Breckon JD, Claytor RP (2011). Adolescent physical activity participation and motivational determinants across gender, age, and race. J Phys Act Health.

[35] Minges KE, Chao A, Nam S, Grey M, Whittemore R (2015). Weight status, gender, and race/ethnicity: are there differences in meeting recommended health behavior guidelines for adolescents?. J Sch Nurs.

[36] Nelson MC, Story M, Larson NI, Neumark-Sztainer D, Lytle LA (2008). Emerging adulthood and college-aged youth: an overlooked age for weight-related behavior change. Obesity.

[37] Steinbeck K, Baur L, Cowell C, Pietrobelli A (2009). Clinical research in adolescents: challenges and opportunities using obesity as a model. Int J Obes.

